# Modulated electro‐hyperthermia induced p53 driven apoptosis and cell cycle arrest additively support doxorubicin chemotherapy of colorectal cancer in vitro

**DOI:** 10.1002/cam4.2330

**Published:** 2019-06-10

**Authors:** Tamas Vancsik, Gertrud Forika, Andrea Balogh, Eva Kiss, Tibor Krenacs

**Affiliations:** ^1^ 1st Department of Pathology and Experimental Cancer Research Semmelweis University Budapest Hungary; ^2^ Institute of Clinical Experimental Research Semmelweis University Budapest Hungary

**Keywords:** caspase dependent apoptosis, colorectal cancer, doxorubicin potentiation, modulated electro‐hyperthermia, p53 and p21^waf1^ upregulation, tumor senescence

## Abstract

**Objective:**

Modulated electro‐hyperthermia (mEHT), a noninvasive complementary treatment of human chemo‐ and radiotherapy, can generate selective ~42°C heat in cancer due to elevated glycolysis (Warburg‐effect) and electric conductivity in malignant tissues. Here we tested the molecular background of mEHT and its combination with doxorubicin chemotherapy using an in vitro model.

**Methods:**

C26 mouse colorectal adenocarcinoma cultures were mEHT treated at 42°C for 2 × 60 minutes (with 120 minutes interruption) either alone or in combination with 1 µmol/L doxorubicin (mEHT + Dox). Cell stress response, apoptosis, and cell cycle regulation related markers were detected using qPCR and immunocytochemistry supported with resazurin cell viability assay, cell death analysis using flow‐cytometry and clonogenic assay.

**Result:**

Cell‐stress by mEHT alone was indicated by the significant upregulation and release of hsp70 and calreticulin proteins 3 hours posttreatment. Between 3 and 9 hours after treatment significantly reduced anti‐apoptotic XIAP, BCL‐2, and BCL‐XL and elevated pro‐apoptotic BAX and PUMA, as well as the cyclin dependent kinase inhibitor p21^waf1^ mRNA levels were detected. After 24 hours, major elevation and nuclear translocation of phospho‐p53(Ser15) protein levels and reduced phospho‐Akt(Ser473) levels were accompanied by a significant caspase‐3‐mediated programmed cell death response. While mEHT dominantly induced apoptosis, Dox administration primarily led to tumor cell necrosis, and both significantly reduced the number of tumor progenitor colonies 10 days post‐treatment. Furthermore, mEHT promoted the uptake of Dox by tumor cells and the combined treatment additively reduced tumor cell viability and augmented cell death near to synergy.

**Conclusion:**

In C26 colorectal adenocarcinoma mEHT‐induced irreversible cell stress can activate both caspase‐dependent apoptosis and p21^waf1^ mediated growth arrest pathways, likely to be driven by the upregulated nuclear p53 protein. Elevated phospho‐p53(Ser15) might contribute to p53 escape from mdm2 control, which was further supported by reduced phospho‐Akt(Ser473) protein levels. In combinations, mEHT could promote the uptake and significantly potentiate the cytotoxic effect of doxorubicin.

## 1 INTRODUCTION

Besides their potential benefits, chemotherapeutics can produce serious and systemic side‐effects including acute gastrointestinal, blood/bone marrow, neurological symptoms, and late adverse effects including cardiomyopathy, infertility, and secondary malignancies.^1^, ^2^ Therefore, any treatment combination which can support the antitumor effect of chemotherapy without increasing the risk of side or adverse effects, or allow drug use at lower concentrations with the same efficiency, would be of great benefit.

Modulated electro‐hyperthermia (mEHT) is a loco‐regional noninvasive complementary of radio‐ and chemotherapy, which has been exploited in these combinations for successfully treating for example human gliomas, soft tissue sarcomas well as cervical, colorectal, and breast adenocarcinomas.^3^, ^4^, ^5^ mEHT uses 13.56 MHz amplitude modulated electric field induced between two plan‐parallel electric condenser plates embracing the tumor area (capacitive coupling).^6^ Elevated glucose uptake and glycolysis (Warburg effect), exploited already in FDG‐PET CT (18‐fluoro‐2‐deoxyglucosepositronemissioncomputer‐tomography),^7^ can result in increased ion concentration and electric conductivity in malignant tumors compared to normal tissues.^8^ These lead to the selective accumulation of electric field to generate ~42°C heat in cancer controlled with the instrument.^9^ Compared to conduction heating hyperthermia, mEHT has been proven significantly more efficient in tumor damage due to its immediate penetration and the synergy between the induced heat and the direct effect of electric field, which can be concentrated in lipid rafts carrying cell membrane receptors.^10^, ^11^ We and other groups have revealed tumor damaging molecular pathways induced by mEHT monotherapy alone using tumor models of diverse histogenesis. Our studies in tumor models confirmed that irreversible heat and cell stress induced by mEHT treatment can lead to programmed tumor cell death (apoptosis) and the release of damage associated molecular pattern signals (DAMP) relevant for inducing an immunogenic cell death (ICD).^12^, ^13^, ^14^ Indeed, a single mEHT shot of C26 colorectal adenocarcinoma grafted into immunocompetent BALB/c mice led to the progressive accumulation of cytotoxic T‐cells and tumor damage suggestive of a secondary ICD response.^12^ Though experimental data support the contribution of mEHT in improving dendritic cell^13^, ^14^ or radiation therapy,^15^ much less information has been collected on mEHT mechanism of action when combined with chemotherapy.

Doxorubicin (Dox), an anthracycline antitumor antibiotic, is frequently used in first‐line chemotherapy, can destruct cancer cells both by preventing DNA repair in proliferating cells and by generating reactive oxygen species (ROS) which can disrupt cell membranes and proteins.^16^ Dox can intercalate DNA strands and interfere with topoisomerase‐II and other DNA‐repair and cell‐cycle control related gene functions involving for example, MLH1, MSH2, and TP53.^17^ ROS can be released when Dox is oxidated to semiquinone, an unstable compound which is then converted back spontaneously to doxorubicin to induce lipid‐peroxidation, membrane disruption, DNA damage, and apoptosis.^18^ Though Dox is a remarkable clinical antineoplastic drug its use carries the risk of cardiotoxicity either through mitochondrial damage by ROS^19^ and/or by interfering with iron regulation, mitochondrial proton pumps, calcium pumps in the sarcoplasmic reticulum, and Na^+^/K^+^ pumps in the cell membranes.^20^

Dox treatment can also result in chemoresistant tumor‐cell populations overexpressing multidrug resistance related xenobiotic transporters including ABCB1 (MDR1, Pgp) and ABCC1 (MRP1) and others (ABCC2, ABCC3, ABCG2, and RALBP1).^20^ Furthermore, this treatment can differentially modify phosphorylated Akt kinase levels depending on the tumor cells type, which play important roles in the tumor cell survival. For example, in OVCAR‐3 and OVCAR‐4 ovarium adenocarcinoma and SKOV‐6 breast adenocarcinoma cell lines Dox treatment can increase Akt phosphorylation on Ser473 linked to HER3 pathway activation.^21^ Akt can also activate XIAP protein and induce caspase‐3, ‐7, ‐9 degradation,^22^ besides promoting p53 inactivation through phosphorylation of the p53 controlling Mdm2 at Ser166 and ‐186.^23^ Since alternating current was shown to increase Dox uptake by tumor cells,^24^ mEHT might be a suitable candidate for attenuating adverse effects of Dox in addition to potentially merging the inherent antitumor effects of these modalities when combined.

In this study we set up an in vitro mEHT treatment model using C26 colorectal adenocarcinoma cell line. First, we validated the reproducibility of cell stress and apoptosis inducing effect of mEHT treatment as published recently using C26 tumors in vivo,^12^ and then extending our focus both to already studied and novel pathways. Having been analyzed the Dox affect alone on C26 cell line, we combined Dox with mEHT treatment to see how they influence each others’ effect. Our results revealed different mechanisms of Dox (dominantly necrosis) and mEHT (mainly apoptosis) therapy, which were significantly added together when they were combined. Our established model can be exploited for future testing of the feasibility of mEHT in combination with chemo‐, radio‐, or targeted therapy modalities in vitro before setting up in vivo tumor models for combined therapy in immunocompetent animals, which best simulate human tumor therapy. Understanding the mechanism of action of mEHT and its interactions with other treatment options in model systems will help better design human combined treatment strategies.

## 2 MATERIALS AND METHODS

### 2.1 Cell culturing

C26 murine colorectal adenocarcinoma cell line (CLS Cell Lines Service GmbH, Eppelheim, Germany, #400156) was grown in RPMI 1640 with 300 mg/L L‐glutamine content (#LM‐R1640, Biosera, Boussens, France) including 10% heat inactivated fetal calf serum (#FB‐1090/500, Biosera) and 80 mg gentamicin (Sandoz GmbH, Basel, Switzerland). Cells were released from sub‐confluent monolayers using 0.25% trypsin and 0.22 mg/mL ethylenediaminetetraacetic acid for 5 minutes, suspended in fresh medium and 4 × 10^5^ cells were grown using 4 mL medium in 60 mm Petri dishes containing a 24 × 40 mm coverslip each.

### 2.2 Combined mEHT and doxorubicin treatment

After 48 hours growth, coverslip cultures were mEHT treated 2 × 30 minutes (with 5 minutes preheating) at 42°C between two plan‐parallel electric condenser plates by using the Lab‐EHY 100 device (Oncotherm Kft, Budaors, Hungary) with a 120 minutes break in between treatments. After the second treatment coverslip‐cultures were put into fresh culture medium. In combined treatment the medium also contained 1 µmol/L Dox at this point. For identifying the optimal Dox concentration 2 × 10^4^ cells/well were treated using 0, 0.01, 0.1, 1, or 10 µmol/L Dox using 96 well plates. Twenty‐four hours post‐treatment resazurin viability assay was performed suggesting that 1 µmol/L drug concentration can lead to sufficient tumor death, which was used in all later treatments either using Dox in monotherapy or in combination with mEHT treatment. Each treatment group included 3‐5 independent experiments.

### 2.3 Gene expression tested with quantitative RT‐PCR

mRNA was extracted 1, 3, 9, and 24 hours after mEHT treatment of cultured tumor cells using RNeasy Mini Kit (#74104, Qiagen, Venlo, Netherlands). Complementary DNA synthesis was done with RevertAid First Standard cDNA Synthesis Kit (#K1622, Thermo Scientific, Waltham MA, USA). QPCR testing of RPLP0 (housekeeping gene), PUMA, BAX, BAK1, XIAP, BCL‐2, BCL‐XL, and P21 gene expression (Table 1; all primer pairs were purchased from Sigma‐Aldrich, St Luis, USA was performed with CFX Connect Real‐Time PCR Detection System (Bio Rad, California, USA) using the SsoAdvanced Universal SYBR Green Supermix (#1725271, Thermo Scientific)) according to the vendors instructions. The fold‐change of the genes of interest relative to RpLp0 was defined as 2^−ΔΔCT ^values.

**Table 1:** Primer sequences used for measuring pro‐ and anti‐apoptotic mRNA levels

Gene	Forward primers (5′‐3′)	Reverse primers (5′‐3′)
BAK‐1	CAGCTTGCTCTCATCGGAGAT	GGTGAAGAGTTCGTAGGCATTC
BAX	AAGCTGAGCGAGTGTCTCCGGCG	GCCACAAAGATGGTCACTGTCTGCC
BCL‐2	CTCGTCGCTACCGTCGTGACTTCG	CAGATGCCGGTTCAGGTACTCAGTC
BCL‐XL	AACATCCCAGCTTCACATAACCCC	GCGACCCCAGTTTACTCCATCC
P21	GCAGAATAAAAGGTGCCACAGG	AAAGTTCCACCGTTCTCGGG
PUMA	TCTATGGGTGGAGCCTCAGT	GAGGGCTGAGGACCCATTAAA
RPLP0	CTCTCGCTTTCTGGAGGGTG	ACGCGCTTGTACCCATTGAT
XIAP	ATGCTTTAGGTGAAGGCGAT	CATGCTGTTCCCAAGGGTCT

### 2.4 Doxorubicin uptake and treatment related tumor cell viability, and cell count

For measuring Dox uptake cultured samples were collected in Fluoroskan Ascent Microplate Fluorimeter (Thermo Scientific) using 530/590 or 570/590 nm (excitation/emission) filter pairs. Cell viability assay was done using a homemade stock solution of 0.3 mg/mL resazurin (#R7017‐5G, Sigma‐Aldrich) dissolved in sterile‐filtered PBS, which was used in 0.03 mg/mL both in treated and control cell culture medium and in plain medium for calculating background intensity. Fluorescent intensity was measured after 1 hour resazurin incubation using the 570/590 nm (excitation/emission) filter pair, since this provided better separation for Dox fluorescence intensity measurements.

### 2.5 Measurement of apoptosis‐necrosis ratio

Apoptotic cell fractions were identified by flow cytometry. Cells were trypsinized 24 hours after treatment, all further steps were done at 0‐4°C to avoid Dox diffusion out of cells. Samples were washed in 2 mL PBS for three times by centrifuging for 5 minutes each time at 500 G. A mixture of 1 µL FITC‐Annexin V stock solution (#640906, Biolegend, San Diego, CA, USA) and 1 µL of 1 mg/mL propidium‐iodide (PI, # P4170, Sigma‐Aldrich) was added to 10^5^ cells in 100 µL PBS. Samples were incubated in dark for 15 minutes, then supplemented with 400 µL Annexin V Binding Buffer (#422201, Biolegend) for the measurement. Argon Ion laser at 488 nm excitation wavelength was used for both fluorochromes. FITC‐Annexin V was measured on FL1 (filter: 530/30 nm), while PI on FL2 channel (filter: 585/42 nm) by counting 2 × 10^4^ events per sample by using flow cytometry (BD FACSCalibur, BD Bioscience, San Jose, USA).

### 2.6 Cell cycle analysis for measuring SubG_1_ fractions

For confirming the termination of apoptosis subG_1_ phase cell fractions were tested using flow cytometry. 10^5^ tumor cells were fixed in 1 mL 70% ethanol at room temperature for 20 minutes then they were kept for at least 30 minutes at −20°C, washed, and resuspended in 250 µL PBS and incubated for 15 minutes with 20 μg/mL RNase (#R6513, Sigma‐Aldrich). Finally, PI stain was applied for 15 minutes and the fluorescence intensity of 2 × 10^4^ events was measured in the FL2 channel.

### 2.7 Polarized membrane staining

Mitochondrial membrane integrity was tested using 3,3′‐Dihexyloxacarbocyanine iodide (DiOC6, # 318426, Sigma‐Aldrich) flow cytometry.^25^ A total of 10^5^ tumor cells washed in PBS were treated for 15 minutes with 10 nM/mL DiOC6 solution made from 1 mmol/L stock solution, which was prepared with absolute ethanol, and 2 × 10^4^ events were measured in the FL1 channel. Both here and at SubG_1_ fraction testing (see above) the BD CellQuest Pro software (BD Bioscience) was used for data analysis.

### 2.8 Immunocytochemistry, hematoxylin‐eosin staining, and image analysis

Cell cultures were fixed in 4% paraformaldehyde/PBS solution for 10 minutes at 4°C then washed three times in PBS. Permeabilization was done using 0.05 mol/L tris‐buffered saline pH 7.4 (TBS) containing 0.3% Tween‐20 (#P9416, Sigma‐Aldrich; 0.3% TBST) for 20 minutes and after washing in 0.1% TBST the samples were used either for hematoxylin‐eosin staining or for immunocytochemistry. For the latter, nonspecific binding sites were blocked for 20 minutes using TBST containing 3% BSA (#82‐100‐6, Merck, Darmstadt, Germany). Rabbit antibodies for calreticulin (1:200, clone: D3E6, #12238), cleaved caspase‐3 (1:100, clone: 5A1E, #9664), hsp70 (1:50, #4872, polyclonal), and phospho(Ser139)‐histone γ‐H2AX (1:150, clone: 20E3, #9718) (all from Cell Signaling, Danvers, MA, USA); and goat polyclonal antibody for p53 (1:350, #AF1355, Bio‐Techne Minneapolis, MN, USA) diluted in 1% BSA/TBST were used for overnight incubations at room temperature. For immunofluorescence Alexa Fluor 546 (orange‐red) coupled anti‐rabbit Ig (1:200) was used for 90 minutes, and cell nuclei were stained blue with 4′,6‐diamidino‐2‐phenylindole (both from Invitrogen/Molecular Probes, Carlsbad, CA, USA). For immunoperoxidase reactions the EnVision polymer‐peroxidase conjugated anti‐rabbit Ig (Dako, Glostrup, Denmark) was used for 40 minutes followed by a DAB chromogen/hydrogen peroxide kit (Leica‐NovoCastra, Newcastle Up‐on‐Tyne, UK). Finally, cell nuclei were counterstained with a hematoxylin, the stained coverslip cultures mounted onto glass slides were digitalized and evaluated using the QuantCenter image analysis software package (3DHISTECH, Budapest, Hungary). The ratio of marker positive cells at different intensity ranges were calculated using the CellQuant module.

### 2.9 Immunofluorescence for flow cytometry

Twenty‐four hours after mEHT treatment supernatants were collected, coverslip cultures were trypsinized and washed in 2 mL PBS by centrifugation at 500 G three times for 5 minutes. Cells were counted in a Bürker‐chamber and used in appropriate amounts for apoptosis‐necrosis and DiOC6 measurements. Fixation in 4% paraformaldehyde/PBS for 30 minutes at 4°C and washing three times in PBS, was followed by permeabilization in 0.2% Tween‐20/PBS for 20 minutes. After repeated centrifugations the supernatants were discarded and 10^5^ cells were immunolabeled for 30 minutes at 4°C by using Alexa Fluor^®^ 488 conjugated rabbit monoclonal phospho‐Akt(Ser473) (1:100, clone: D9E, #4060) and cleaved caspase‐8 (Asp387) (1:800, clone: D5B2, #8592) (both from Cell Signaling) and mouse monoclonal phospho‐p53(Ser15) (1:50, clone:16G8, #9235) antibodies diluted in 1% BSA/PBS. For detecting unlabeled rabbit antibodies an Alexa Fluor 488 conjugated anti‐rabbit Ig (1:200) (Invitrogen/Molecular Probes) was used for 30 minutes. All samples and negative controls were washed in PBS by three times centrifugation and measured in the FL1 channel. Both here and at apoptosis‐necrosis ratio testing the FlowJo v10 software (FlowJo LLC, Ashland, Oregon, USA) was used for data analysis.

### 2.10 Clonogenic assay

Twenty‐four hours after treatment, cells were trypsinized, and 500 cells/well were cultured in 6‐well plates using three experimental parallels. Ten days after plating the cell medium was discarded and the wells were washed three times in PBS then fixed in 4% buffered formaldehyde for 30 minutes. After washing again in distilled water samples were dried out then stained with 0.1% crystal violet for 20 minutes washed again and cell colonies were counted manually.

### 2.11 Statistics

Each result was calculated from at least three experimental and three biological parallels. Statistical analysis for parametric variables was done with the independent two‐sample t‐test. For nonparametric variables the Mann‐Whitney *U* test was used (SPSS15.0, Chicago, IL, USA). Statistical significance was declared at *P*‐values of *P < 0.05, **P < 0.01, and ***P < 0.001.

## 3 RESULTS

### 3.1 mEHT monotherapy induced cell stress, apoptotic signaling, and programmed cell death

**Figure 1:**
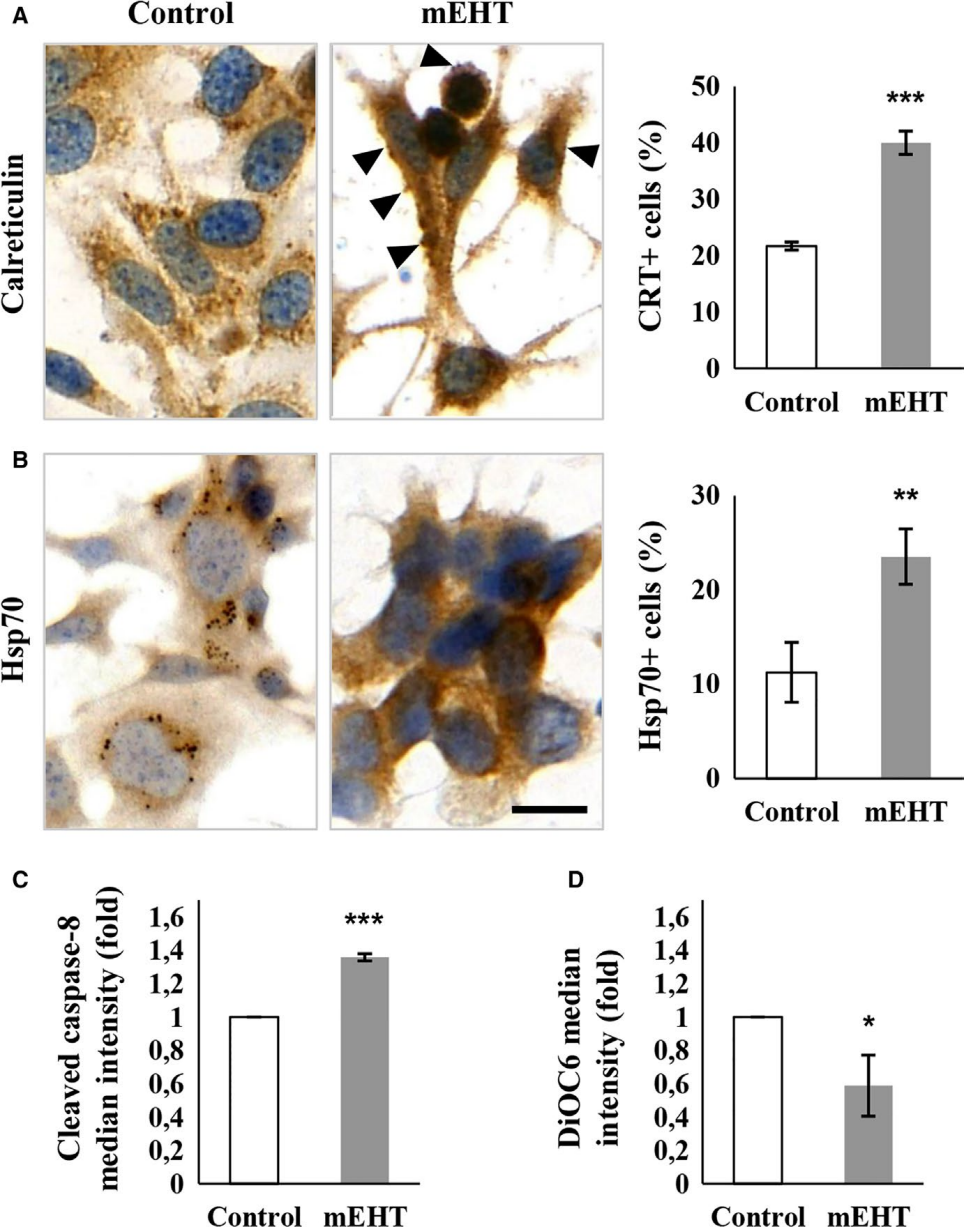
Signs of significant cell stress in C26 tumor cells 24 h after mEHT treatment. Cytosolic release and cell membrane translocation of calreticulin with positive membrane blebs (arrowheads) (A). Elevated cytoplasmic hsp70 reaction released from paranuclear vesicles (B). Scale bar: 20 µm. Significantly increased cleaved caspase‐8 levels in tumor cells (C) and reduced DiOC6 uptake by mitochondrial membranes (D) measured using flow cytometry indicate the induction of both the intrinsic and the extrinsic programmed cell death pathways, respectively. *P < 0.05; **P < 0.01; ***P < 0.001

Similar to our earlier in vivo studies,^10^ cell‐ and heat‐stress as well as apoptosis related markers showed major increase in protein level accompanied by programmed cell death response in subconfluent C26 colorectal adenocarcinoma cultures 24 hours after 2 × 30 minutes mEHT monotherapy controlled at 42°C. Significant upregulation and relocalization of calreticulin from the endoplasmic reticulum to the cytoplasm and cell membranes were observed in treated cultures (40.02 ± 2.05) compared to the untreated controls (21.70 ± 0.69) (Figure 1A). Calreticulin positive cell membrane blebbing regions suggested the release of this antigen embraced within small extracellular vesicles. Also, the proportion of tumor cells showing elevated hsp70 levels with diffuse pattern, instead of concentrating in the endoplasmic reticulum‐Golgi region, increased from 11.26 ± 3.18 to 23.52 ± 2.92 as a result of mEHT treatment (Figure 1B). Furthermore, the median intensity of the cleaved caspase‐8 labeled cell fraction suggesting the activation of the extrinsic apoptotic pathway was also increased to 1.36 ± 0.02‐fold (Figure 1C), while the polarized membrane‐staining of DiOC6 indicating intact mitochondrial membranes, was significantly reduced after mEHT (58.87 ± 18.36%) compared to control cultures (Figure 1D).

**Figure 2:**
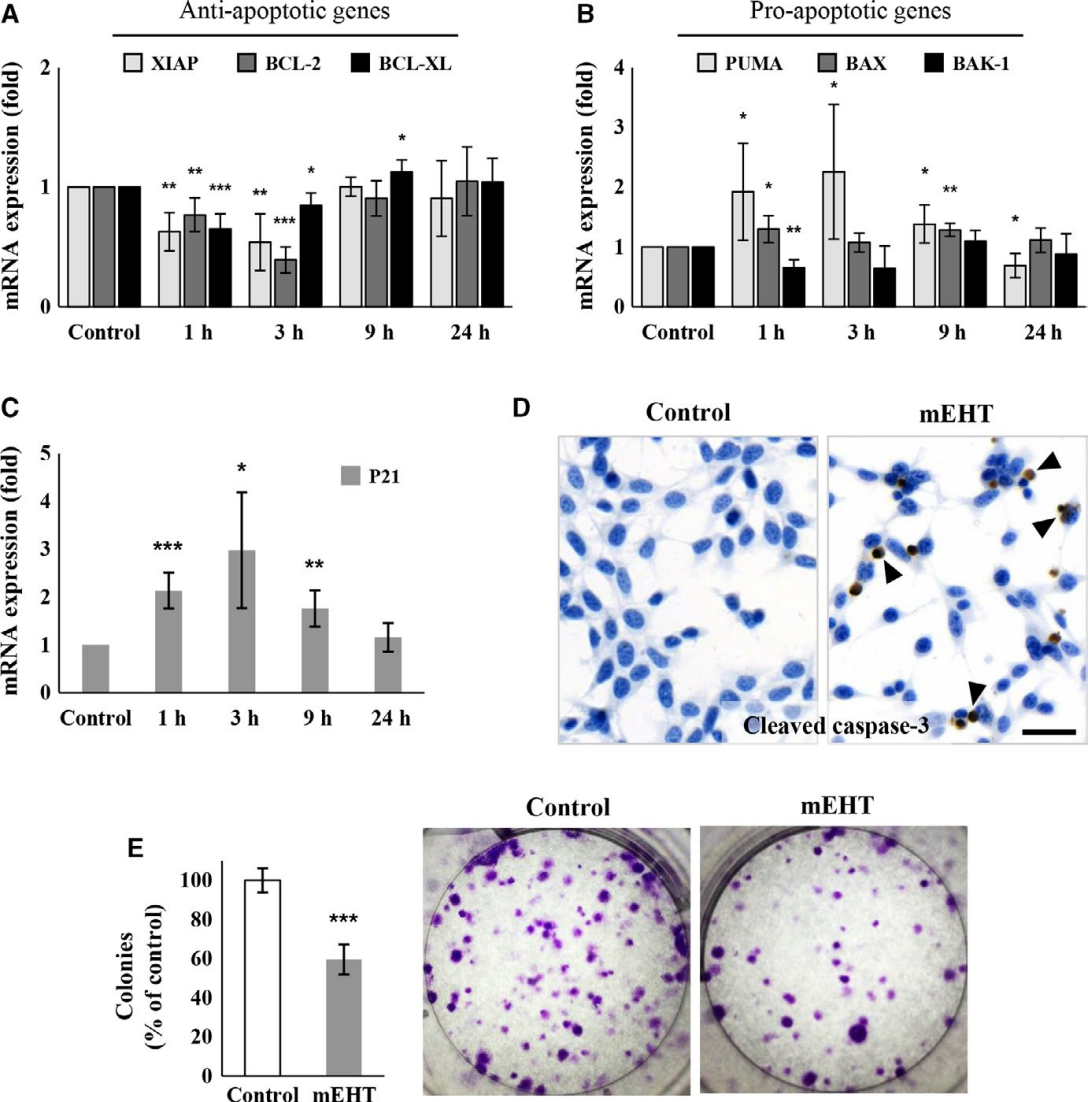
Expression of apoptosis regulation related genes in C26 tumor cells after mEHT treatment. Significant reduction in the anti‐apoptotic XIAP, BCL‐2, BCL‐XL mRNA levels 1 and 3 h posttreatment (A). Elevated pro‐apoptotic PUMA mRNA levels at 1, 3, and 9 h, and BAX levels at 1 and 9 h after mEHT (B). Similarly increased temporal pattern of P21 mRNA levels to that of PUMA (C). In line with the apoptosis‐promoting mRNA profile, cleaved caspase‐3 protein expression (arrowheads) was significantly elevated 24 h after treatment as tested with immunocytochemistry (D). Scale bar: 100 µm. Significantly reduced colony‐forming tumor progenitor‐cell populations 10 d after mEHT treatment (E). *P < 0.05; **P < 0.01; ***P < 0.001

Apoptosis and cell‐cycle regulation related gene expression was studied at the mRNA level to see how early response elements react to therapy. mEHT monotherapy induced a major mRNA fold‐decrease in the anti‐apoptotic BCL‐2, BCL‐XL, and XIAP transcripts both after 1 hour (0.77 ± 0.14, 0.65 ± 0.13, and 0.63 ± 0.16 respectively) and 3 hours (0.39 ± 0.11, 0.85 ± 0.1 and 0.54 ± 0.24, respectively) post‐treatment, then returned to the control levels between 9 and 24 hours (Figure 2A). mRNA levels of the pro‐apoptotic BAX showed moderate but prolonged increase which was significant at 1 hour (1.3 ± 0.23 fold) and 9 hours (1.28 ± 0.11 fold) posttreatment (Figure 2B). The pro‐apoptotic PUMA (Figure 2B) and the cyclin dependent kinase inhibitor P21 transcript levels also revealed significant increase in 1 hour (1.92 ± 0.81, 2.13 ± 0.38 fold), 3 hours (2.25 ± 1.12, 2.97 ± 1.21 fold), and 9 hours (1.38 ± 0.31, 1.76 ± 0.38 fold) posttreatment (Figure 2C). These changes were accompanied by the significant elevation of the cleaved/activated caspase‐3 protein positive tumor cell fraction in the treated cultures compared to the controls (Figure 2D).

In clonogenic assay colony formation from tumor progenitor/stem cell clones was significantly reduced after mEHT monotherapy (59.55 ± 7.73%; *P* < 0.001) (Figure 2E).

### 3.2 Combination of mEHT and doxorubicin treatments

**Figure 3:**
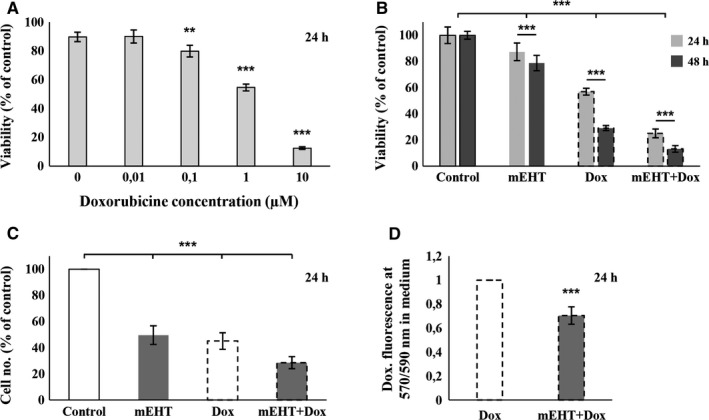
Comparison and combination of doxorubicin (Dox) and mEHT treatments in C26 tumor cell cultures. 1 µmol/L Dox, reducing cell viability (resazurin assay) by ~40%, was chosen for further investigations (A). Progressively and significantly reduced tumor cell viability (B) and numbers (C) 24 and 48 h after mEHT, Dox and combined (mEHT + Dox) treatments. Enhanced effect of the combined treatment might be due to the reduced Dox concentration (elevated Dox uptake) in the culture supernatants (D). **P < 0.01; ***P < 0.001

Serial dilutions of Dox were tested to optimize its therapeutic concentration in C26 cultures. Accordingly, treatment using 1 µmol/L Dox concentration led to an LD60 value as measured with resazurin cell viability assay after 24 hours incubation (Figure 3A), which was then applied in the relevant treatment protocols. In comparative testing, mEHT reduced tumor cell viability to 87.35 ± 6.36%, Dox treatment to 56.92 ± 2.62 while their combination resulted in only 25.00 ± 3.31% surviving tumor cells at 24 hours (Figure 3B). Forty‐eight hours after mEHT treatment cell viability was further reduced to 78.82 ± 5.84%, 29.06 ± 1.89%, and 13.17 ± 2.48%, respectively (Figure 3B). After 24 hours the remaining tumor cell numbers also showed strong correlation with the resazurin assay particularly after combined treatment. This was also significant after mEHT monotherapy 49.61 ± 7.12% while Dox monotherapy revealed only a loose correlation with the resazurin test (45.14 ± 6.31%) (Figure 3C). Dox concentration in the culture medium was also tested based on its fluorescence intensity using 570/590 nm excitation/emission filter pair. When combined with mEHT Dox levels in culture supernatants were reduced to 0.70 (±0.07)‐fold of those of Dox monotherapy suggesting a promoted drug uptake (Figure 3D). Dox at 1 µmol/L concentration, both alone and in combination with mEHT, completely killed tumor progenitor cell clones (not shown).

### 3.3 Treatment related Akt and p53 activation

**Figure 4:**
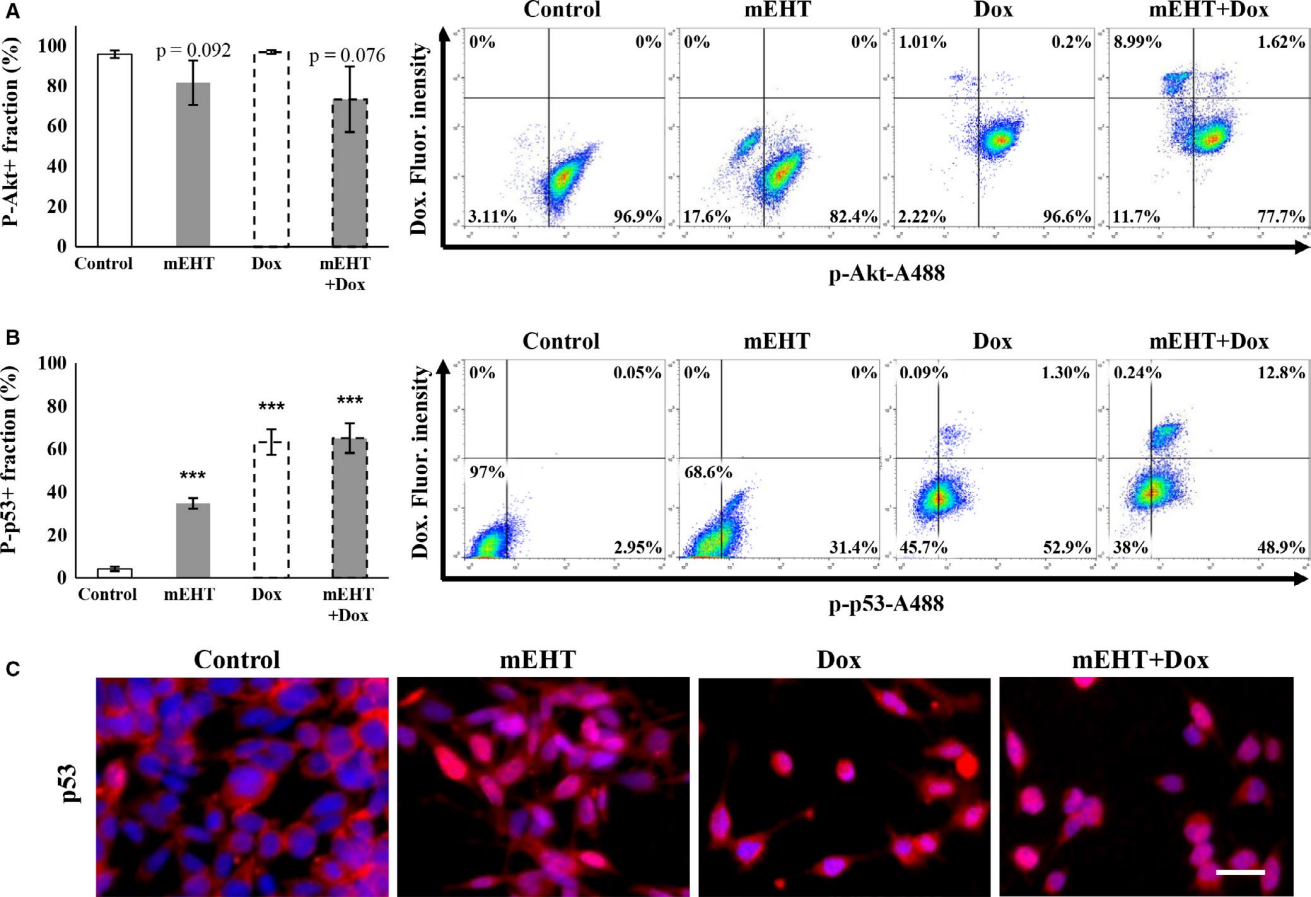
Comparison of phospho‐Akt and ‐p53 levels 24 h after treating cultured C26 tumor cells. Reduced phospho‐Akt(Ser473) kinase positive cell populations after mEHT and mEHT + Dox treatments (A). Significantly elevated post‐treatment phospho‐p53(Ser15) levels showing the same highest values after Dox and the combined treatments (B). Increased cytoplasmic to nuclear relocalization of phospho‐p53(Ser15) indicate p53 activation (C). Scale bar: 100 µmol/L. ***P < 0.001

Survival related Akt kinase activation was measured through the phospho‐Akt(Ser473) positive cell fractions, which showed reduction (from 95.97 ± 1.89%) after both mEHT (85.01 ± 10.06%, *P* = 0.061) and Dox + mEHT treatments (76.33 ± 15.64%, *P* = 0.059) (Figure 4A). At the same time, the activated tumor‐suppressor phospho‐p53(Ser15) protein positive cell populations were significantly increased from 4.27 ± 0.99% base level up to 34.7 ± 2.4% after mEHT, to 63.3 ± 5.96% after Dox and to 65.13 ± 6.95% after combined treatments (all *P* < 0.001) (Figure 4B). With immunocytochemistry, elevated number of tumor cells showing nuclear translocation of the p53 protein was detected in the treated cultures indicating the stabilization and activation p53 protein (Figure 4C).

### 3.4 Mechanism of tumor cell death induced by doxorubicin and mEHT

**Figure 5:**
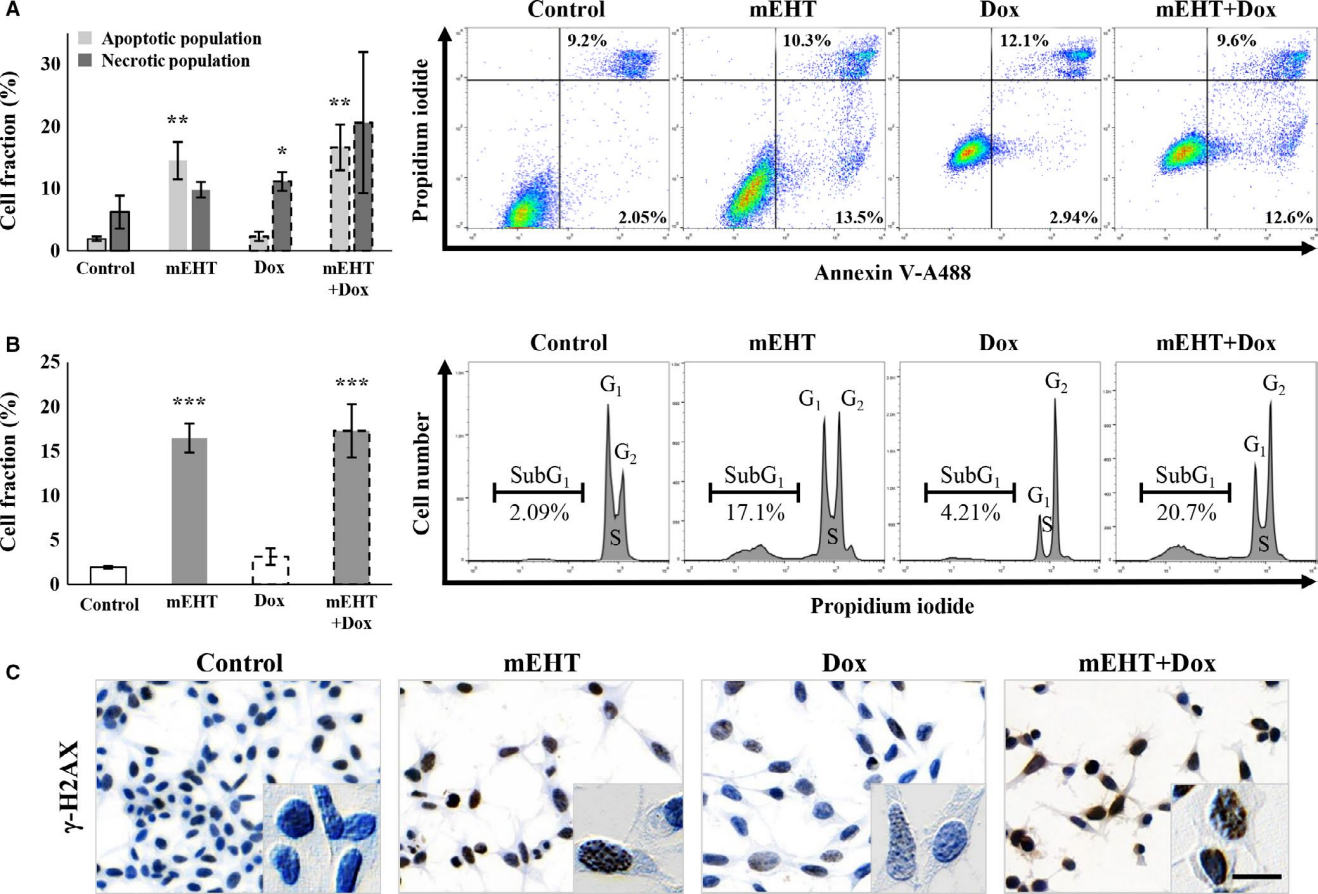
Comparison of the ratio of cell and DNA damage 24 h after treating cultured C26 tumor cells. Significantly elevated apoptotic cell fractions after mEHT and necrotic cell fractions after Dox treatments and their additive, merged effect after combination (mEHT + Dox) therapy (A). Significant increase in subG_1_ phase cell fractions both after mEHT and combined treatments refer to the apoptosis‐related DNA damage (B), where DNA double‐strand breaks were indicated by upregulated γ‐H2AX granular positivity (brown) in cell nuclei using immunocytochemistry (C). Scale bar: 100 µmol/L. *P < 0.05; **P < 0.01; ***P < 0.001

In line with our earlier in vivo results^10^ mEHT monotherapy induced a significant increase in the apoptotic tumor cell fractions in vitro (14.53 ± 2.99%) compared to the untreated cultures (1.94 ± 0.36%). At the same time, apoptosis was not significant (2.31 ± 0.73%) after Dox treatment (Figure 5A). This finding was further supported by the combined mEHT + Dox treatment resulting in only similar proportions of apoptotic cell populations (16.67 ± 3.69%) to that of mEHT monotherapy (Figure 5A). The necrotic cell populations detected also in the control cultures (6.24 ± 2.64%) increased more after Dox monotherapy (11.18 ± 1.50%) that after mEHT (9.84 ± 1.25%) which were added together after combined treatment (20.63 ± 11.36%) (Figure 5A).

### 3.5 Treatment‐related nuclear damage DNA double‐strand breaks

The proportion of cells with apoptosis‐related nuclear damage was grown significantly from 1.92 ± 0.16% to 16.47 ± 1.64% after mEHT, to 3.13 ± 0.94 after Dox and to 17.27 ± 2.99% after combined mEHT + Dox treatments (Figure 5B). DNA double strand‐breaks indicated by the increased intensity and granularity of the γ‐H2AX immunoreaction in tumor cell nuclei, was also detected at high levels both after mEHT monotherapy and after combined mEHT + Dox therapy compared to controls (Figure 5C).

### 3.6 Treatment related cell cycle shift and tumor colony‐formation

**Figure 6:**
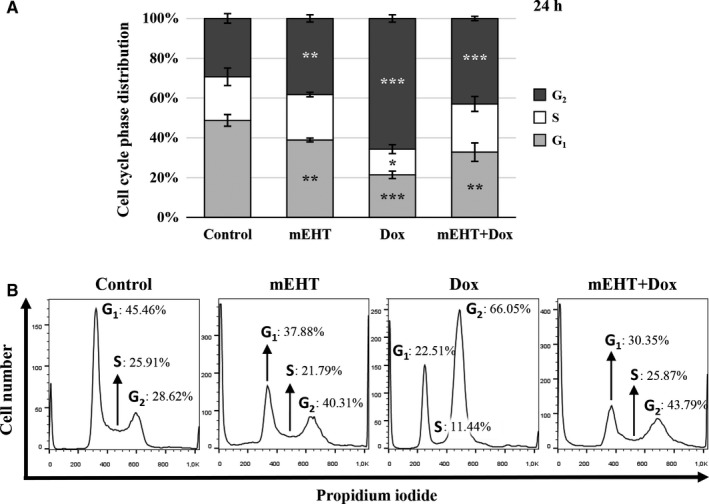
Summary graph (A) of flow cytometry analysis (B) of the treatment‐related cell cycle fractions. Significant but less G_2_ phase cell cycle arrest seen 24 h after mEHT than after Dox treatment, where the sizes of G_1_ and G_2_ populations in the combined (mEHT + Dox) group show about the averages of the single treatments. *P < 0.05, **P < 0.01, and ***P < 0.001

G_1_ phase cell populations in the cell cycle were significantly reduced to 38.89 ± 0.97% after mEHT, to 21.41 ± 1.84% after Dox and to 32.81 ± 4.67% after mEHT + Dox treatments compared to the control cultures 48.81 ± 2.91% (Figure 6A‐B). S‐phase cell populations showed decrease only after Dox monotherapy, while G_2_ phase cell fractions were increased after both monotherapies, to 38.24 ± 1.81% after mEHT, and to 65.69 ± 1.82% after Dox, but only to 42.97 ± 1.05% upon mEHT + Dox treatments compared to the control (29.37 ± 2.4%) levels (Figure 6A‐B).

## 4 DISCUSSION

Earlier studies confirmed that mEHT treatment used as a complementary of chemo‐ and radiotherapy of human cancer can induce tumor destructions by itself both by provoking apoptosis through irreversible heat and cell‐stress, and by affecting cell membrane‐fluidity and targeting dielectric membrane receptor molecules concentrated in lipid rafts.^11^, ^26^ These effects and the increased loco‐regional blood perfusion by mEHT added to those of the combination partner are likely to contribute to the improved treatment efficacy of these combinations.^3^, ^4^, ^5^ However, the molecular mechanisms of interactions between mEHT and chemotherapy, which would help designing better treatment protocols, still need to be clarified. Here we set up an in vitro test system for rapid dissection of mEHT effects using C26 a mouse colorectal cancer cell line, which we used earlier to demonstrate the mEHT treatment related immunological events in vivo.^12^ The similar tumor destructive mechanisms revealed in the two models validated that our in vitro model is appropriate for feasibility testing of mEHT in combinations with other treatment modalities worth for further in vivo analysis. Combination of mEHT with Dox chemotherapy resulted in an additive tumor cell destruction and control by merging efficient apoptosis and necrosis induction of mEHT and Dox, respectively, and the cell cycle arrest contributed by both components.

In monotherapy, in line with our in vivo allograft results, mEHT provoked cell‐ and heat‐stress in cultured C26 tumor cells indicated by the upregulation and cytoplasmic translocation of hsp70 and calreticulin proteins.^12^ In culture, the latter was obviously enriched in tiny cell membrane blebs of damaged tumor cells suggesting its release within extracellular vesicles possibly including exosomes that may form protective shells around their content.^27^, ^28^ Hsp70 and calreticulin are part of the damage associated molecular pattern (DAMP) signaling, which after release can augment the receptor mediated uptake, processing, and presentation of tumor antigen to promote antitumor immune response.^29^, ^30^, ^31^, ^32^ Indeed, we confirmed this by showing that after a single mEHT treatment CD3+ (very rarely FoxP3 positive) T‐cells and S100 positive antigen presenting cells showed progressive accumulation accompanied by an ongoing tumor damage.^12^ After in vitro mEHT treatment, the upregulation of pro‐apoptotic (PUMA and BAX), the downregulation of anti‐apoptotic gene (XIAP, BCL‐2 and BCL‐XL) transcripts (between 1 and 3 hours) followed by the significant elevation of cleaved/activated caspase‐3 protein positive cultured tumor cells (after 24 hours) were consistent with caspase dependent apoptosis.^33^, ^34^, ^35^ The increased, cleaved/activated caspase‐8 protein levels and damaged mitochondrial membrane permeability (reduced DiOC6 absorption), suggested the activation of both the extrinsic and the intrinsic programmed cell death pathways.^25^, ^36^ However, less subG_1_ phase fraction indicating DNA fragmented apoptotic cell population was measured that it was expected from the increase of annexin‐V positive (early sign of apoptotic membrane damage) cell fraction implying that cell cycle progression was also hindered by mEHT treatment.^37^ This was supported by p21 transcript upregulation, we found between 1 and 9 hours post mEHT treatment, encoding the cyclin dependent kinase inhibitor p21^waf1^ protein that can mediate cell cycle arrest (and senescence).^38^

Both caspase dependent apoptosis through the upregulation of pro‐apoptotic and downregulation of anti‐apoptotic mediators (see above) and cell cycle arrest through inducing P21 expression are likely to be orchestrated by the upregulated nuclear phospho‐p53(Ser15) protein.^39^, ^40^ Activation of p53 could be induced both by heat and cell‐stress and the DNA double‐strand breaks as the latter was indicated by the accumulation of nuclear γ‐H2AX protein after mEHT treatment.^41^ Phosphorylation of p53 protein at Ser15, we detected here, is known to prevent p53 ubiquitination by mdm2 and thus can promote p53 functions.^23^ Akt can also interfere with p53 activation and inhibit its mitochondrial function including apoptosis.^23^, ^42^, ^43^ Thus reduced survival‐related phospho‐Akt(Ser473) levels we measured after mEHT treatment can further support the role of p53 in tumor control, since activated Akt could have promoted mdm2 control of p53 and the anti‐apoptotic XIAP for interfering with caspase‐3 activity.^44^, ^45^, ^46^

Dox monotherapy of cultured C26 colorectal cancer cells was feasible at 1 μmol/L concentration relevant for reducing tumor cell viability by 40%.^47^, ^48^ Under comparable conditions this decrease significantly exceeded that of mEHT, but instead of inducing apoptosis, necrosis was the dominant cell damage mechanism by Dox with significantly less DNA double‐strand breaks measured than after mEHT. However, Dox contributed more significantly to p53 activation and killing of tumor (stem) progenitor cell clones than mEHT. Comparative analysis of cell cycle fractions also revealed a major G_2_ phase arrest after Dox treatment and less but still significant G_2_‐arrest after mEHT compared to untreated cultures.

Combination of Dox with mEHT treatments resulted in an additive reduction in tumor cell viability (almost reaching synergy level) and number as well as cumulated the apoptotic, necrotic, and the whole lost/damaged cell fractions. Our results also suggest that mEHT can promote the uptake of Dox by tumor cells. This combination may further raise the sensitivity and/or reduce the therapeutic concentration and side effects of Dox, in agreement with the observation that alternating current (that also delivers mEHT) can enhance Dox uptake.^24^

In conclusion, here we set up an in vitro mEHT treatment model of C26 mouse colorectal cancer useful for feasibility studies of the molecular background of combining mEHT with other treatment modalities, starting with Dox chemotherapy. Besides validating similar cell stress and programmed cell death pathways in this model to our allograft system using the same cell line, we also extended our focus to refine cell damage pathways involved in the mEHT effect. Our results show that mEHT monotherapy can induce irreversible cell stress both through caspase‐dependent apoptosis and p21^waf1^ mediated cell cycle arrest, which are likely be driven by p53 activation. Elevated phospho‐p53(Ser15) and reduced phospho‐Akt(Ser473) we measured are known to promote p53 escape from mdm2 control. In combination with Dox, mEHT promoted the uptake and significantly potentiated tumor destruction and control by Dox through merging efficient apoptosis and necrosis induction by mEHT and Dox, respectively, and the cell cycle arrest contributed by both treatments. This model can serve for pilot testing of mEHT combinations prior to comprehensive investigations of allografted C26 cells using immune competent animals.

## CONFLICT OF INTEREST

The authors declare no conflict of interest in this work.
